# A Review on Bioactivities of Perilla: Progress in Research on the Functions of Perilla as Medicine and Food

**DOI:** 10.1155/2013/925342

**Published:** 2013-11-11

**Authors:** Miho Igarashi, Yoshifumi Miyazaki

**Affiliations:** Center for Environment, Health and Field Sciences, Chiba University, Kashiwanoha 6-2-1, Kashiwa, Chiba 277-0882, Japan

## Abstract

Perilla is a useful pharmaceutical and food product and is empirically consumed by humans. However, its properties have not been evaluated extensively. In this review, we summarize the progress made in research, focusing on the bioactivities of perilla. There are many *in vitro* and animal studies on the cytostatic activity and antiallergic effects, respectively, of perilla and its constituents. However, its influence on humans remains unclear. Hence, investigating and clarifying the physiological effects of perilla and its constituents on humans are imperative in the future to adhere to the ideals of evidence-based medicine.

## 1. Introduction

Perilla is a type of aromatic vegetables. Asian people preferred consuming it since ancient times. It was used to treat fish and crab poisoning symptoms according to Chinese classics [1]. This information passed on from China throughout Asia; thereafter, perilla is used as a traditional medicine and functional food in Asia.

In traditional medicine, aromatic substances are often used to treat mental stress. Perilla is included as one of them, and it is also used in combination with other aromatic oriental medicines that are referred to as Kampo medicines (hereafter “Kampo”). Hangekobokuto (Chinese name: Banxia-Houpo-Tang), Kososan (Chinese name: Xiang-Su-San), and Suyu-Jiaonang (SYJN) are representative Kampo, and these are used to treat depression-related diseases and asthma [2].

It is reported that Hangekobokuto can ameliorate sleep choking syndrome [3], swallowing reflex [4, 5], and panic disorder [6] in humans and has antidepressant effects in mice [7]. Ito et al. [8] reported that Kososan exhibited antidepressant-like effects in murine forced swimming model, and Suyu-Jiaonang (SYJN) demonstrated antistress effects in rats [9]. However, the importance and role of perilla in these Kampo medicines remain to be clarified.

In Japan, these Kampo have been used as medicine for a long time and are now approved and used as general medicines. In recent years, Kampo has attracted attention as an alternative medicine among the foreign countries, including those in Europe and the United States, and the World Health Organization (WHO) announced that they will add a chapter on traditional medicines, including Japanese Kampo, in the eleventh edition of the International Classification of Diseases (ICD-11) in 2015. Accordingly, scientific investigations of the effects of Kampo are advancing [4–9]. Nonetheless, for many years, Kampo has been used in traditional Asian medicines to treat physical conditions such as stress and asthma.

We review the use of bioactive compounds from perilla varieties as medicines and foods to prevent and ameliorate illnesses.

## 2. Methodology

All published reports on physiological functions of perilla were extracted from the PubMed database.

## 3. Perilla as Food and KAMPO

Two types of perilla, (*Perilla frutescens* (L.) Britton var.* crispa* (Thunb.) H. Deane f. *purpurea* (Makino) Makino and *Perilla frutescens* (L.) Britton var.* crispa* (Thunb.) H. Deane *viridis crispa*), are commonly added to food in Japan. In addition, perilla oil is extracted from *Perilla ocymoides *(*Perilla frutescens* (L.) Britton var.* frutescens*) for consumption in Asia [11] (Table 1).

In Japanese pharmacy, leaves and branches of *Perilla frutescence* Britt. var. *acuta* Kudo and *Perilla frutescence *Britt. var.* crispa* Decaisne as Perillae Herba [10] (Table 1) are defined as medicines.  Table 2 shows the main constituents of perillae herba [10].

## 4. Bioactivity of Perilla

### 4.1. Effects of the Kampo Perillae Herba

Perillae herba has been used as an oriental medicine for many years in Asia and has been passed on through generations by experience. In Japan, evident effects of Kampo containing perillae herba have been recently reported. However, the effects of the perillae herba itself are not stated clearly. Perillae herba is included in Japanese Kampo medicines such as Hangekobokuto (Chinese name: Banxia Houpu; [3–6, 12–15]), Kososan (Chinese name: Xiang-Su-San; [8]), and Saibokuto (Chinese name: Chai-Pu-Tang) for the treatment of cough and stress symptoms [2]. Perillae herba is also found in Chinese Suyu-Jiaonang (SYJN), which is also used to treat mental conditions [9, 16].

### 4.2. Effects of Perilla Decoctions

Antiallergic effects of perilla decoctions have been demonstrated on mice. In these studies, perilla decoctions partly controlled IgA nephropathy [17] and type I allergies [18]. It is thought that these effects are caused by rosmarinic acid. In addition, perilla decoctions demonstrated suppressive effects on mesangioproliferative glomerulonephritis in rats [18].

In HIGA mouse model (IgA renal damage), perilla decoctions alleviated IgA nephropathy through adjustments of the mucous membrane. Moreover, rosmarinic acid, present in high quantities in perilla, was found to be a constituent of perilla decoctions, and it is believed that the maximum effect of perilla decoction is caused by rosmarinic acid [17].

The effects of rabbit anti-rat thymocyte serum were examined in a BALB/c mouse model of mesangioproliferative glomerulonephritis, and suppressive effects were observed [19].

The positive effects of perilla decoctions were observed in ddY mice with type I allergies [18]. After the oral administration of  500 mg/kg perilla decoction and the relative amount of rosmarinic acid in a mouse model with ear passive cutaneous anaphylaxis (PCA), allergic reactions were inhibited. Because the inhibition rates of perilla decoctions and rosmarinic acid were approximately equal, it was believed that the effect of perilla decoction depended on rosmarinic acid [18, 20]. However, comparisons of the effects of rosmarinic acid with perilla decoctions indicate additional therapeutic constituents in perilla.

### 4.3. Effects of Perilla Extract

In cultured murine vascular smooth muscle cells, perilla extract has been shown to induce NO production, indicating that perilla may be useful for the prevention of vascular diseases such as arteriosclerosis [21]. In addition, perilla extract increased restraint and induced cell death in human hepatoma HepG2 cells [22]. Moreover, flow cytometry and DNA microarray experiments revealed significant apoptosis and time-dependent regulation of apoptotic genes, respectively, in cells treated with perilla leaf extracts.

However, in this study the experiment was conducted *in vitro* with a high dose of the perilla extract; therefore, it is unclear if perilla extract is effective *in vivo*.

### 4.4. Effects of Perilla Oil

Perilla oil contains large quantities of alpha-linolenic acid, an essential fatty acid, which decreases the risk of cardiovascular diseases [23]. Several *in vitro*, animal, and human nutritional studies have focused on the effects of perilla oil and have compared it with those of other oils. Perilla oil increases glucose-6-phosphatase activity, improves membrane stability [24], lowers plasma triacylglycerol levels [25], and controls liver fatty acid composition [26].

Perilla oil, which is rich in n-3 polyunsaturated fatty acids, regulates brown and white adipose tissue metabolism in a manner different from that of safflower and fish oils and contributes to physiological activities that prevent body fat accumulation, regulate glucose metabolism in rats [27], and control serum lipid concentrations [28]. Perilla oil rich in alpha-linolenic acid is effectively desaturated and elongated to form eicosapentaenoic acid (EPA) and docosahexaenoic acid (DHA, [29]). In addition, in light- and dark-discrimination learning tests, perilla oil-administrated senescence-accelerated mice (SAMP8) group exhibited higher discriminability than safflower oil-administrated group [30]. Moreover, serum lipids in the perilla oil-administrated SAMP8 group had a significantly greater ratio of apolipoprotein A-I (ApoA-I) to ApoA-II than those in the safflower-oil administered group [31]. Perilla oil was not as effective as soybean oil in preventing minor recurrent aphthous stomatitis; however, *in vivo*, *in vitro*, and lifestyle studies report positive effects of perilla oil on minor recurrent aphthous stomatitis [32].

## 5. Effects of Perilla Constituents

Perilla contains several essential oils, including (−)-perillaldehyde, (−)-perillylalcohol, (+)-limonene, alpha-pinene, and trans-shisool. Moreover, perilla contains the purple pigments shisonin and cyanin. Other constituents of perilla include rosmarinic acid, adenine, and arginine [10].

### 5.1. Effects of Perillaldehyde

In contrast with the Kampo cinnamaldehyde, perillaldehyde elicited antidepressant-like effects on the olfactory nervous system in mice that were subjected to chronic weak stress and a forced swimming test [33]. Antidepressant-like effects were evaluated by measuring the duration of immobility in the forced swimming test. Decreased duration of immobility was observed after 9 days of perillaldehyde inhalation. In contrast, the odorant cinnamaldehyde from cinnamon bark failed to decrease the duration of immobility. This antidepressant effect of perillaldehyde was abolished in mice induced with anosmia by zinc sulfate, confirming the olfactory mode of action.

In Wistar rat aortas, vasodilatation following inhibition of Ca channels was ameliorated by perillaldehyde, with dose-dependent aorta extensibility, and relaxation with 0.01–1 mM treatments with prostaglandin F2*α* or norepinephrine [34]. In addition, these effects were unchanged by treatment with NG-nitro-L-arginine methyl ester or removal of aorta endothelium. Therefore, it was suggested that perillaldehyde directly affects vascular smooth muscle. Furthermore, vasodilatation was not inhibited by the *β*-adrenergic-receptor blocker propranolol, the phosphodiesterase inhibitor theophylline, the delayed rectifier K^+^ channel blocker tetraethylammonium chloride, or the ATP-sensitive K^+^ channel blocker glibenclamide. In contrast, perillaldehyde caused vasodilatation of contracted aortas via a mechanism involving Ca^2+^ transport out of the cell. Perillaldehyde caused slight vasodilatation of Ca^2+^-ionophore (A23187) contracted aortas but inhibited highly concentrated K^+^-mediated vasodilatation. Given the predominance of voltage-dependent Ca^2+^ efflux, it was suggested that perillaldehyde acted as a Ca^2+^ channel inhibitor for vasodilatation.

In cultured human liver microsomes, perillaldehyde slightly inhibited the hydroxylation of bupropion (antidepressant) by the enzyme CYP2B6 [35] and inhibited *Candida albicans* [36]. In addition, in 293 TRPA1-HEK cells, perillaldehyde activated the transient potential A1 receptor [37], suggesting that this discovery will enable future analysis of taste properties.

Perillaldehyde inhibited the proliferation of human squamous cell carcinoma of the tongue (BroTo) and human lung adenocarcinoma (A549) [38] and demonstrated anticancer activity in PC12 cells (rat pheochromocytoma cell line [39]). Moreover, Masutani et al. [40] reported the activation of the Nrf2-Keap1 system by perillaldehyde. Furthermore, antibacterial actions (using air washers) against floating microbes were reported [41].

Perillaldehyde was found to preserve fruits and promote the antioxidant activity of blueberries [42] and Chinese bayberries [43]. Hence, these natural products may be helpful in the maintenance of crop quality and security. However, further research is required to examine the influence of perillaldehyde on flavour, texture, and harvest parameters of crops.

### 5.2. Perillyl Alcohol

Perillyl alcohol is the subject of only few *in vitro* studies. In these studies, perillyl alcohol induced cell cycle arrest and cell death in BroTo; [38], and A549 [38]. Perillyl alcohol also had inhibitory effects on HCT116 cells (human colon cancer cell line; [44]) and caused dose-dependent inhibition of mammary tumor cell proliferation.

### 5.3. Perillic Acid

Perillic acid has been shown to inhibit proliferation of HCT116 cells (human colon cancer, [44]), mammary tumor cells [45], and PC12 cells (rat pheochromocytoma; [39]), indicating anticancer properties.

### 5.4. Rosmarinic Acid

Rosmarinic acid is a constituent of rosemary; therefore, there are several reports on this constituent of perilla.

It was reported that rosmarinic acid inhibits seasonal allergic rhinoconjunctivitis in humans [46]. This double-blind study indicated that oral supplementation with rosmarinic acid is an effective intervention for patients in the age group of 21–53 years with mild seasonal allergic rhinoconjunctivitis. In this 21-day experiment, rosmarinic acid significantly increased responder rates for itchy nose, watery eyes, itchy eyes, and total symptoms and decreased neutrophil and eosinophil numbers in nasal lavage fluid. The authors suggest that rosmarinic acid may reduce treatment costs for allergic diseases.

The absorption, metabolism, and urinary excretion of rosmarinic acid after a single dose of perilla extract were determined in six healthy men (mean age 37.2 ± 6.2 years and mean body mass index 22.0 ± 1.9 kg/m^2^; [47]). In this study, rosmarinic acid was absorbed, conjugated, and methylated following intake, and a small proportion of rosmarinic acid was degraded into various constituents, including conjugated forms of caffeic acid, ferulic acid, and m-coumaric acid, which were rapidly excreted in urine.

Several studies have examined the effects of rosmarinic acid on allergic reactions in mice. In nasal mucosa of ovalbumin-sensitized BALB/c mice, rosmarinic acid and 30% ethanol extract powder of perilla reduced the number of ear and eye rubs, the levels of IgE and histamine in the serum, inhibited protein and mRNA expression of interleukins (IL)-1*β*, IL-6, and tumor necrosis factor-*α*, and inhibited cyclooxygenase-2 protein expression and caspase-1 activity [48]. Moreover, rosmarinic acid blunted nuclear factor-kappa B (NF-*κ*B)/Rel A and caspase-1 activation. These results suggested that both rosmarinic acid and 30% ethanol extract powder of perilla alleviate allergic inflammatory reactions such as allergic rhinitis and allergic rhinoconjunctivitis.

Consistent with this report, rosmarinic acid ameliorated type I allergies in ddY mice [49] and inhibited ear-passive cutaneous anaphylaxis (PCA) reactions more effectively than the modern antiallergic drug tranilast. 

In HIGA mice that spontaneously develop high levels of serum immunoglobulin A (IgA) and mesangial IgA deposition, rosmarinic acid suppressed serum IgA levels and may suppress IgA nephropathy [18].

In forced swimming tests, rosmarinic acid produced an antidepressant-like effect, at least in part via the proliferation of new-born cells in the dentate gyrus of the hippocampus [50]. Rosmarinic acid also reduced the defensive freezing behaviour of mice exposed to conditioned fear stress [51]. The authors suggest that investigating the mechanisms of these effects may help to explain the pathophysiology of such affective disorders, which would facilitate new therapeutic strategies and future development of novel anxiolytic and/or antidepressive drugs.

In other studies, rosmarinic acid demonstrated anticarcinogenic effects in a murine two-stage skin model [52]. This paper concluded that rosmarinic acid contributes to the anticarcinogenic effects of perilla via two independent mechanisms: by inhibiting inflammatory responses and potent superoxide scavenging activity. Furthermore, rosmarinic acid ameliorated LPS-induced liver injury in D-GalN-sensitized mice [53].

*In vitro*, rosmarinic acid inhibited mesangial-cell proliferation [54] and adriamycin-induced apoptosis in H9c2 cardiac muscle cells [55]. Moreover, Kim et al. suggested that rosmarinic acid should be viewed as a potential chemotherapeutic that inhibits cardiotoxicity in adriamycin-exposed patients. Indeed, rosmarinic acid significantly prevented 6-OHDA-induced cell viability reduction [56], indicating a potential as a chemotherapeutic agent for the treatment of Parkinson's disease. Finally, rosmarinic acid inhibited the formation of reactive oxygen and nitrogen species in RAW264.7 macrophages [44].

## 6. Other Constituents

Perillaketone has been shown to activate cloned TRPA1 channels *in vitro* [37]. Bassoli et al. [37] suggested that these data may explain the taste properties of the plant at the molecular level and identify it as an interesting target for its culinary and pharmaceutical applications.

Carvane was shown to inhibit the transformation of *Candida albicans* [36] and also inhibited type I allergies in ddY mice [18].

Luteolin inhibits inflammation and allergic responses *in vivo* and *in vitro* [57], and the mechanisms of relaxant action were elucidated in isolated guinea pig tracheas [58].

The perilla constituent 1,2-Di-O-alpha-linolenoyl-sn-glycerol, which was extracted from the local variety, kida-chirimen shiso, inhibited superoxide generation [59].

## 7. Discussion

In this review, we were not able to find investigations on dietary perilla, though numerous studies revealed bioactive constituents of perilla. 

We searched the literature from medicine, pharmacology, and nutritional sciences to clarify the functions of perilla. We found very few studies of perilla on humans but many *in vivo* and *in vitro* investigations of its biological properties. Whereas dietary effects of perilla remain unknown, perilla oil is considered a high quality oil in nutritional sciences, which are concerned with lifestyle-related diseases.

According to numerous *in vivo* and *in vitro* studies, perilla and its constituents have anticancer, antiallergy, and antidepressant properties, and the biological activities of perilla and its constituents are increasingly well characterised (Table 3). Although there are several *in vitro* studies demonstrating anticancer properties, studies conducted *in vivo* are very few. Hence, further research is required to examine the effects of using perilla as a food and medicine in daily life.

Among active constituents of perilla, perillaldehyde and rosmarinic acid have received the most attention, suggesting that these compounds are important constituents. Perillaldehyde contributes to aroma and accounts for approximately 50% of the oil refined from perillas. Therefore, it is considered important to examine the effects of this compound on stress; as for Kampo and food, its pleasant aroma is well known. However, only one paper considers bioactivity from aroma alone [31]. As rosmarinic acid is a constituent of both perilla and rosemary, scientific data is available for rosmarinic acid than for the other perilla compounds. Consequently, its effects on allergies are well characterised in humans [46].

In this review, we have summarized the current progress in perilla research. Although we found abundant *in vitro* and animal studies, we could not find a human study regarding the traditional theory on the effects of Kampo aroma on stress and depression. Investigating and clarifying the physiological effects of perilla and its constituents on humans are imperative in the future to adhere to the ideals of evidence-based medicine (EBM).

## 8. Conclusion

Animal and *in vitro* studies abundantly demonstrate the effects of perilla and its constituents; however, their influence on humans is nearly unknown. In contrast, perilla has already been used as a form of oriental medicine and as food. Investigating and clarifying the physiological effects of perilla and its constituents on humans are imperative in the future to adhere to the ideals of EBM.

## Figures and Tables

**Table 1:** Types of a perilla used for food and medicinal purposes [10, 11].

	Scientific name
Food	*Perilla frutescens *(L.) Britton* crispa *(Thunb.) H. Deane f. *purpurea *(Makino) Makino
	*Perilla frutescens *(L.) Britton* crispa *(Thunb.) “*Viridis crispa*”
	*Perilla frutescens *(L.)* frutescens *

Medicine	*Perilla frutescens *Britton var. *acuta *Kudo
Perillae Herba	*Perilla frutescens *Britton* crispa *var. Decaisne

**Table 2:** The main constituents in perillae herba [10].

	Compounds
Essential oil	(−)-perillaldehyde, (+)-perillalcohol, (+)-limonene, alpha-pinene, and trans-shisool

Pigment	Cyanin and shisonin

Other compounds	Rosmarinic acid, adenine, and arginine

**Table 3:** The main effects of perilla.

Product studied	Main effects	Type of study	Number of papers
Perilla decoction	Type I antiallergic effect Mouse ear passive cutaneous anaphylaxis (PCA) reaction IgA nephropathy in HIGA mice Decreased proliferation of mesangial cells	Animal	5

Perilla extract	Inhibits growth and induces NO production in vascular smooth muscle cells	*In vitro *	2

Perilla oil	Protective effects against recurrent aphthous stomatitis (−)	Human	1
	Fatty acid synthesis, improved learning in the Sidman active avoidance task	Animal	8
	Cytostatic activity	*In vitro *	1

Perillaldehyde	Antidepressant-like effect through regulation of the olfactory nervous system	Animal	2
	Vasodilatory effect Cytostatic activity and so on	*In vitro *	6

Perillyl alcohol	Cytostatic activity	*In vitro *	2

Perillic acid	Cytostatic activity	*In vitro *	2

Rosmarinic acid	Seasonal allergic rhinoconjunctivitis inhibition, absorption, metabolism, degradation, and urinary excretion	Human	2
	Antiallergic effects against type I allergies Antidepressant-like effect Anticarcinogenic effect	Animal	8
	Cytostatic activity	*In vitro *	4

Caffeic acid	Absorption, metabolism, degradation, and urinary excretion	Human	1
	Antidepressant-like effect	Animal	1
